# Mental health literacy: knowledge of depression among undergraduate students in Hanoi, Vietnam

**DOI:** 10.1186/s13033-018-0195-1

**Published:** 2018-04-24

**Authors:** Quynh Chi Nguyen Thai, Thanh Huong Nguyen

**Affiliations:** Hanoi University of Public Health, Hanoi, Vietnam

**Keywords:** Mental health literacy, Undergraduate students, Depression, Vietnam

## Abstract

**Background:**

Mental health literacy (MHL) refers to an individuals’ knowledge and beliefs about mental disorders that aid their recognition, management, and prevention. This study aims to investigate the MHL of depression among public health and sociology undergraduate students in Hanoi, Vietnam.

**Methods:**

A cross-sectional survey was carried out from May to September 2015. Data was collected using an anonymous, self-administered questionnaire distributed to 350 undergraduate students (213 public health majors; 137 sociology majors). Questions about MHL of depression were adapted from the Australian National Survey of Mental Health Literacy and Stigma. Question topics included recognition of depression, help-seeking intentions, first-aid support, and knowledge about interventions to help people with depression. Chi squared tests were conducted to compare proportional statistics across groups for multiple measures.

**Results:**

With regard to recognition of mental disorders, 32.0% of the respondents used the accurate label “depression” for the vignette. Among those who correctly identified depression, 82.1% would seek help. The corresponding statistic was 81.1% from those who did not recognize depression. Both groups would seek help from counselor, psychologist, family members, and close friends. First-aid support suggested by the respondents in both groups were informal sources (*to listen to her problem in an understanding way, to encourage her to be more physically active*, etc.). The interventions considered most helpful by the respondents were self-help strategies such as *learning how to relax*, *getting physically active*, doing *exercise early in the morning*, and *reading a self*-*help book*. *Joining a group of individuals with similar problems* was chosen to be a helpful intervention among those who did not recognize depression (p < 0.001), but those who correctly identify depression believed that people with depression should be admitted to hospital for psychiatric treatment (p < 0.05).

**Conclusion:**

There is a need for education about MHL of depression among undergraduate students in Vietnam. The training can focus on symptoms of depression, appropriate help-seeking intentions, and first-aid support relevant to the Vietnamese context.

## Background

According to the World Health Organization (WHO), there are approximately 450 million people with mental disorders and more than that with mental health problems. Mental disorders account for 13% global burden of disease, and its prevalence appears to be increasing [1]. The results from Global Burden of Disease study in 2015 showed that neurological conditions in Vietnam contributed 4.56% of total DALYs [2]. Mental health problems often arise for the first time in adolescents or young adults [3–5] and affect more than one in four young people globally. In Vietnam, the prevalence of general mental health problems ranges from 8 to 29% for children and adolescents. This percentage among young people, including undergraduate students, is higher at about 25–60% [6, 7].

Depression is a common and serious mental health problem. According to WHO [8], there were at least 350 million people living with depression globally. Depression often starts at a young age, can be long-lasting or recurrent, and when most severe, can lead to suicide [9]. In Vietnam, depressive disorders have the second highest prevalence among mental disorders [10] with about 36,000–40,000 people losing their lives to depression per year [11]. Depression is treatable, but fewer than half of those affected in the world (in many countries, less than 10%) receive the care and support they need [9]. Shortage of resources, lack of psychiatric services, social stigma, and lack of mental health literacy (MHL) constitute major barriers in accessing treatment for mental disorders in general and for depression in particular [9, 12, 13]. Healthcare services in Vietnam are organized around a 4-tier system (central, province, district, commune) with two major types of services: community-based and hospital-based [10]. More mental health services are provided in hospitals than in community, but follow-up usually occurs at community general practice [14].

The term MHL was first used in 1997 by Jorm to describe “knowledge and beliefs about mental disorders which aid their recognition, management or prevention” [15]. This definition emphasizes the role of recognition of mental health problems and help-seeking for management and prevention by young people and people close to them, e.g. family members, teachers, and friends. The MHL research to date has demonstrated that many young people do not seek help or postpone help-seeking due to various personal and structural barriers such as fear of stigma and discrimination associated with depression; inability to recognize symptoms of the problems; lack of knowledge about the availability of help; lack of appropriate responses from both peers and adults [16, 17].

Studies assessing MHL of depression of the population have been done in some countries. Jorm and his colleagues, in a study published in 1997, found that 39% (n = 1010) of Australians aged 18–74 could correctly recognize depression [18]. Another survey 15 years later among 1016 Australians aged 15 and older found that 73.7% of the respondents could recognize depression [19]. In a survey among 1004 Canadians aged 18–64, divided into two groups: 18–24 and 25–64, Marcus and Westra found that the ability to recognize depression among the two age groups were 80 and 79% respectively [20]. MHL was also studied in some Asian countries showing a lower percentage of respondents who could correctly recognize depression. Jingyi Wang et al. found that 34.6% (n = 1.953) of Shanghai residents selected the correct answer [21]. A study among a multiracial population in Singapore (2016) showed that 55.2% of this population (including Chinese, Malay, Indian and others) could correctly name a vignette as depression [22].

MHL of depression focused on young people has been studied elsewhere. Coles et al. found that 40% of high school students aged 14–19 (n = 1104) in New York, USA could correctly recognize depression symptoms described in a vignette [12]. A study in UK [23] found that among 1125 young people aged 16–24, 41.8% was able to correctly name a vignette as depression [17]. Reavley et al. estimated that 74% of higher education students in Australia could recognize depression in a vignette [3]. Sayarifard et al. [24] carried out a survey among 324 medical sciences students in Iran and the results showed only 35.6% of the participants selected the correct answer. In Sri Lanka, Amarasuriya et al. [25] examined depression literacy among 4671 undergraduates and only 17.4% of the respondents were able to recognize depression. Because mental health problems often arise at young age, such studies are needed for potential intervention to improve mental health for young people [26]. In Vietnam, there have been some studies estimating prevalence of depression [11, 27]. To the best of our knowledge, there is however no publication that describes young people’s knowledge of mental health. We aim to investigate the recognition of depression, help-seeking intentions, and knowledge to support people with depression among undergraduate Vietnamese students in Hanoi. Findings from our study can be used to inform interventions aiming to promote understanding of MHL of depression and improving mental health.

## Methods

### Study setting and design

This was a cross-sectional study conducted at four universities in Hanoi, Vietnam: (1) Hanoi School of Public Health; (2) Faculty of Public Health, Hanoi Medical University; (3) Faculty of Sociology, University of Social Sciences and Humanities; (4) Faculty of Sociology, Academy of Journalism and Communication. Public health students were selected from the first two institutions and sociology/social sciences students came from the other two schools. These universities were chosen so that comparison could be made between students of different majors.

### Study sample

The survey was carried out between May and September 2015. We began with a convenience sample of 1160 undergraduate students receiving 3 vignettes (anxiety disorders, depression, and schizophrenia) and their corresponding questionnaire in classroom setting. The depression vignette was randomly distributed to 350 students. More information on participant recruitment is presented below.

### Research instruments

A structured questionnaire consisted of two parts: the MHL of depression and socio-demographic information. MHL of depression was assessed using a questionnaire adapted from the Australian National Survey of Mental Health Literacy and Stigma [19]. The survey started with a vignette of a 20-year-old female student experiencing depression, following with questions in four areas: recognition of depression (10 items); help-seeking intentions (9 items); knowledge of first-aid support (9 items); and knowledge of intervention (12 items). The vignette was described as follows.

“*Linh is a 20*-*year*-*old student who has been feeling unusually sad and miserable for the past several weeks. She is tired all the time and has trouble sleeping at night. Linh doesn’t like eating and has lost weight. She can’t keep her mind on her studies and even day*-*to*-*day tasks seem too much for her*”.

This MHL instrument has been used in many studies in different countries. Permission to use the MHL questionnaire was granted by Jorm and his team. The questionnaire was translated into Vietnamese. We piloted the questionnaire with 10 students from public health and 10 from sociology/social sciences to test if the questions were clearly written and receive appropriate answers (face validity). Aside from that, the questionnaire was reviewed by two mental health experts in Hanoi, first one from the National Institute of Mental Health (Bach Mai Hospital) and the other one from the Hanoi National University. Feedback was used to modify the vignette and improve some items in the questionnaire (content validity) [28]. We also tested the internal consistency and reliability of the scale, using Cronbach’s Alpha.

To assess *recognition of depression* from the vignette, the following question was asked: “In your opinion, what is going on with Linh?”. The response format was multiple choice and the answers were: “Attention-deficit hyperactivity”, “Cancer”, “Anxiety”, “Depression”, “Schizophrenia”, “Stress”, “Epilepsy”, “Other (explain)”, “There’s nothing wrong with her”, and “I don’t know”. The correct answer was “Depression”.

To assess *help*-*seeking intentions*, the participants were asked “If your friends have the same problem as Linh, would you intend to do something to help?”. The answers were “Yes”, “No”, and “I don’t know what to do”. The subsequent questions were about potential helpers: “General practitioner/family doctor”, “Teacher”, “Counselor”, “Helpline”, “Psychologist”, “Close family member”, “Close friend”, “Linh has to deal with the problem herself”. For each potential helper, respondents could check one of the four following options: “helpful”, “harmful”, “neither”, and “don’t know”. Cronbach’s Alpha for this scale was 0.69.

To assess *knowledge of first*-*aid support* for people with depression, the following actions were described: “Listen to her problem in an understanding way”; “Talk to her firmly about getting her act together”; “Suggest her seeking professional help”; “Make an appointment for her to see a GP”; “Suggest her have a few drinks to forget about her problem”; “Rally friends to cheer her up”; “Keep her busy to keep her mind off the problems”; “Encourage her to become more physically active”; “Ignoring her until she gets over it”. For each action the response options were: “helpful”, “harmful”, “neither”, and “don’t know”. Cronbach’s Alpha for this scale was 0.61.

Finally, to assess *knowledge of interventions*, the following statements were presented: “Listed are different activities that could help Linh. Circle the option that best reflects your opinion of activity”. The following list was then presented: “Becoming more physically active”; “Learning how to relax”; “Getting acupuncture”; “Getting up early each morning to do exercise”; “Getting counseling”; “Looking for online information to learn about the problem”; “Reading a self-help book on the problem”; “Joining a group of people who have similar problem”; “Going to a local mental health service”; “Being admitted to hospital for psychiatric service”; “Using alcohol to relax”; and “Smoking to relax”. For each option, participants had to circle one of the four options: “helpful”, “harmful”, “neither”, and “don’t know”. Cronbach’s Alpha for this scale was 0.62.

Since MHL consists of multiple dimensions, the level of MHL should not be simply assessed by a single dimension such as the recognition of mental health problem. In order to properly assess an adequate level of MHL, a composite variable was created combining two important dimensions of the MHL construct: recognition of mental health problem, and help-seeking intention. The justification for choosing these two particular dimensions of the MHL construct was based on the definition of MHL [15]. As previously mentioned, the main features of MHL are knowledge and beliefs about mental disorders which aid their recognition, management or prevention [15]. Therefore, a combination of correct identification of the mental health problem and intention to seek help was defined as having an adequate MHL level [29].

### Data collection and statistical analysis

Students were recruited in a classroom setting. Each student was given an envelope which contained the study information sheet, the consent form, and the questionnaire. They were asked to read the study information sheet, signed the consent form, if they agreed to participate in the study, and then answered the questionnaire. Before collecting the data, we had informed the students that they either could stay inside or outside if choosing not to participate in the survey. At the time the completed questionnaire was collected, we clearly stated again that participation in the survey was voluntary. This information was also included in the consent form. The response rate was 100%. After the survey was administered in each university, the questionnaires were checked for completeness. All of the 350 completed questionnaires were included in the analysis. Statistical software SPSS 20 was used for statistical analyses. We calculated the frequencies of multiple variables. Chi squared tests were conducted to test for statistical differences between the two groups of students.

## Results

Among 350 students who completed the questionnaire, 213 (60.9%) were public health students and 137 (39.1%) were sociology/social science students. The majority of respondents were female (76.6%). Most students were living with housemates (51.4%), with parents (35.1%), and the rest were living with relatives, by themselves, with acquaintances or with their partners. The age of respondents ranged from 19 to 26 years with a mean age of 20.7 (SD = 1.3) (see Table 1 for more details).

**Table 1:** Sociodemographic characteristics of respondents (n = 350)

	n	%
Age [mean (SD)]	20.7 (1.3)	
19–20	164	46.9
21–22	165	47.1
23–26	21	6.0
Gender		
Male	82	23.4
Female	268	76.6
Living with		
Parents	123	35.1
Housemates	180	51.4
Partners	2	0.6
Acquaintances	6	1.7
Relatives	18	5.1
Alone	13	3.7
Others	8	2.3

### Identification of depression

**Fig. 1:**
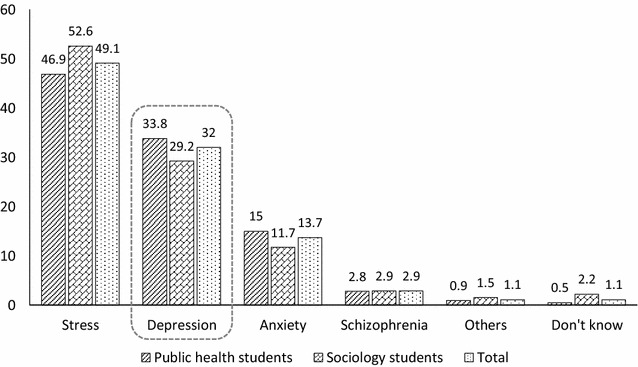
Identification of mental health issues from the vignette

Figure 1 shows the responses given by the participants to the question “In your opinion, what is going on with Linh?”. “Stress” was the most common answer in both groups of students (49.1%). The percentage of students who correctly identified the problems as depression was 32.0% (n = 112).

As also shown in Fig. 1, 33.8% (n = 72) of public health students and 29.2% (n = 40) of sociology students selected the correct label of “depression” for the vignette. There was no significant difference between the two groups in recognition of the problem (*χ*^*2*^ = 4.12; *p* = 0.53). In addition to “depression”, the most frequent responses were “stress” (public health: 46.9%; sociology: 52.6%) and “anxiety” (public health: 15%; sociology: 11.7%). Since there are no statistically significant differences between these two majors, we combined them in our analysis in the next part.

### Help-seeking intentions

Among 350 respondents, 112 students (32.0%) correctly recognized depression from the vignette. When asked if they would go for help if their loved ones/friends had a problem similar to the person in the vignette, 81.4% of the respondents said they would do so, 6.6% would not, and 12.0% did not know what to do.

For MHL level, 92 (26.3%) respondents could be classified as adequate MHL level by the aforementioned definition (correct identification and intention to seek help).

Table 2 reports opinions about the most frequently mentioned help-seeking intentions from 350 respondents, divided into two groups: (1) group 1—students who recognize the vignette as depression; (2) group 2—student who did not recognize the vignette as depression. Students in group 1 were likely to seek help from informal sources such as counsellor (91.9%), psychologist (90.1%), close family members and close friends (85.7%). These persons were also mentioned as the most helpful sources for help-seeking from those in group 2; however, there were no statistically significant differences between the two groups.

**Table 2:** Percentage (and 95% CI) of respondents rating person, first-aid support and interventions that are “helpful” for Linh’s problem

	Recognize depression (group 1) (n = 112)	Not recognize depression (group 2) (n = 238)	Total (n = 350)
	% 95% CI	% 95% CI	%
Person			
Counselor	91.9 (85.1–95.8)	84.0 (78.7–88.1)	86.6
Psychologist	90.1 (82.9–94.5)	86.1 (81.0–89.9)	87.4
Close family member	85.7 (77.8–91.1)	88.2 (83.4–91.7)	87.4
Close friend	85.7 (77.8–91.1)	87.8 (82.9–91.4)	87.1
Mental health professional	68.7 (59.4–76.7)	62.1 (55.8–68.1)	64.3
General practitioner/family doctor	55.3 (45.9–64.4)	49.5 (43.2–55.9)	51.4
Helpline	54.4 (45.0–63.5)	50.8 (44.4–57.1)	52.0
Psychiatrist	54.4 (45.0–63.5)	46.6 (40.3–53.0)	49.1
Teacher	47.3 (38.1–56.6)	44.9 (38.7–51.3)	45.7
Dealing with her problem on her own	20.5 (13.9–29.1)	23.1 (18.1–28.9)	22.3
First-aid support			
Listen to her problem in an understanding way	97.3 (91.8–99.1)	95.7 (92.3–97.7)	96.3
Encourage her to be more physically active	86.6 (78.8–91.8)	81.5 (76.0–85.9)	81.3
Suggest her seek professional help	73.2 (64.1–80.6)	66.3 (60.1–72.1)	68.9
Make an appointment for her to see the GP	69.6 (60.3–77.5)	63.0 (56.6–68.9)	65.1
Rally friends to cheer her up	66.0 (56.6–74.3)	66.8 (60.5–72.5)	66.3
Suggest she have a few drinks to forget about her problems	57.1 (47.6–66.0)	67.2 (60.9–72.9)	64.4
Keep her busy to keep her mind off the problems	25.0 (17.7–33.9)	23.1 (18.1–28.9)	23.7
Ignoring her until she gets over it*	2.67 (0.85–8.10)	5.88 (3.5–9.72)	4.9
Talk to her firmly about getting her act together	1.78 (0.43–6.99)	1.26 (0.40–3.86)	1.4
Interventions			
Learning how to relax	92.8 (86.2–96.4)	94.9 (91.2–97.1)	94.3
Becoming more physically active	86.6 (78.8–91.8)	77.1 (71.5–82.2)	80.3
Receiving counselling	82.1 (73.7–88.2)	76.4 (70.6–81.4)	78.3
Getting up early each morning to do exercise	72.3 (63.1–79.9)	67.6 (61.3–73.3)	69.1
Going to a local mental health service	63.3 (53.9–0.71)	51.2 (44.8–57.6)	55.1
Reading a self-help book on the problem	57.1 (47.6–66.0)	59.2 (52.8–65.3)	58.6
Looking up a website giving information about the problem	55.3 (45.9–64.4)	56.3 (49.8–62.5)	56.0
Joining a group of people who have similar problems***	45.5 (36.4–54.9)	49.1 (42.8–55.5)	48.0
Being admitted to hospital for psychiatric services*	32.1 (24.0–41.4)	26.4 (21.2–32.4)	28.3
Getting acupuncture	19.6 (13.2–28.1)	15.9 (11.8–21.2)	17.1
Using alcohol to relax	7.14 (3.57–13.7)	7.14 (4.47–11.2)	7.1
Smoking to relax	3.57 (1.32–9.25)	5.46 (3.18–9.21)	4.9

* p < 0.05; *** p < 0.001

Health professionals—such as mental health professional, GP/family doctor, psychiatrist—were also mentioned as helpful, but not as highly rated as informal sources of support. There were still 20.5% of the respondents in group 1 and 23.1% of group 2 believed that Linh should deal with her problem on her own.

### First-aid support

To evaluate this component, a list of actions that students might have to support Linh was provided (Table 2). The actions that respondents in both groups considered most helpful were *listen to her problem in an understanding way (96.3%), encourage her to become more physically active (81.3%), suggest her seek professional help (68.9%), and make an appointment for her to see the GP (65.1%)*. However, these differences have no statistical significance (p > 0.05). Group 2 chose *ignoring her until she gets over it* as a first-aid action with higher percentage than group 1 (χ^2^ = 8.11, p < 0.05).

### Knowledge of interventions

When being asked about the interventions for people with depression like Linh, participants in both groups identified the most helpful ones as (Table 2): *learning how to relax (94.3%)*, *becoming more physically active (80.3%)*, *receiving counselling (78.3%)*, *getting up early each morning to do exercise (69.1%)*, *going to a local mental health service (55.1%)* with higher percentage among students in group 1 than those in group 2. However, these differences have no statistical significance. More respondents in group 2 believed in *reading a self*-*help book* and *looking up a website giving information about the problem* than those in group 2. There was also a low percentage of participants thought that *using alcohol to relax (7.1%)* and *smoking to relax (4.9%)* were helpful interventions for people with depression. 49.1% of the respondents in group 2 and 45.5% in group 1 believed that a helpful intervention for Linh’s problem was *joining a group of people who have similar problem* (χ^2^ = 28.66, p < 0.001). More students in group 1 than in group 2 thought that people with depression should be *admitted to hospital for psychiatric service* (χ^2^ = 8.05, p < 0.05).

## Discussion

Recognition of depression is a factor that can facilitate help-seeking [30]. The percentage of young Vietnamese people who could correctly detect depression is lower (32%) than that of developed countries [12], but higher than that of some other developing country [25]. Our result also showed a majority of respondents recognized the vignette as “stress” (49.1%), suggesting that the ability to identify mental health problems among undergraduate students needs to be improved.

Depression often starts at an early age and can be long lasting [9]. As Jorm and his colleagues have pointed out, good MHL in young people and their key supporters may lead to better outcomes for those with mental disorders, either by facilitating early help-seeking by young people themselves, or helping adults to identify early signs of mental disorder and to seek help for the young person [5]. As shown in Lam’s study [29], correct identification of depression and intention to seek help can be used to assess the level of MHL [29]. The low level of correct identification of depression in this study points to the need to improve both knowledge on disorder symptoms and the awareness of resourceful locations to seek help for people with depression.

Even though many respondents were unable to recognize the problem as depression, the majority of respondents (94.8%) did identify that Linh’s problem was a common mental health problem—stress, depression or anxiety. A very small proportion of respondents thought she had schizophrenia (2.9%) or unspecified disorder (1.1%). Only 1.1% of students chose “don’t know”.

It is not surprising that a substantial proportion of the respondents (81.4%) were willing to help. This result is consistent with that reported by Reavley [3] stating that regardless of the proper identification of the disorder, they understood the importance of the need to seek help. This finding indicates young people’s willingness to help those with depression, despite whether they can correctly recognize the specific problem.

In this study, participants showed confidence in informal sources such as family members and close friends when needing help. The bonding with families and friends as well as social network supports in Vietnam is typically strong. It seems people can seek help from their loved ones or from their social network whenever they need. Professional help from formal sources such as mental health professionals, general practitioners/family doctors, and psychiatrists, in this study was not deemed necessary as found in other studies [23, 24]. This is understandable in the context of Vietnam, where most people know very little about what roles mental health professionals and psychiatrists play. Moreover, there is still stigma in the community regarding mental health problems. This might have driven people’s decision to not see a mental health specialist.

Our results showed a high proportion of respondents identified the problem as one of the common mental health problems (stress, depression, anxiety) and they also rated a counselor or a psychologist as a helpful source of intervention. This means our respondents believed in the role of a counselor and a psychologist to help people with common mental health problems, including depression. In Vietnam, however, most psychologists are not practitioners. They mostly work in research or teaching environment. Only some provide counseling through helpline centers. Therefore, respondents’ choice of “talking to a counselor or a psychologist” may be referred to professional help from medical setting.

Telephone helplines provide easy access for those seeking mental health counselling. In Hanoi, there are several helplines that provide psychological counseling. These helplines are open 24/7 assisting people who need help any time. However, most young people are either unaware of helpline services or doubtful of their usefulness. This discrepancy is similar to a finding from Debra Rickwood’s study in Australia (2005) that when young people are seeking help, they want to talk to a professional whom they can trust and that person can maintain confidentiality [31].

In terms of knowledge about first-aid support, among the proposed possible actions that participants could potentially take to support people with depression, the option of “listening to the person who needs help” was chosen by a large percentage of respondents. This action is consistent with the guidelines of mental health first aid [32]. A majority of participants considered it useful to encourage individuals with mental health issues to become more physically active. They believed that the distraction may lead a person to forget about his/her mental health problem temporarily. Depression is a treatable disease; talking with people we trust is a first step towards recovery from depression [9]. WHO encourages people with symptoms of depression to consult professionals or recognize depression among family and friends and encourage them to seek help because depression can lead to suicide [9]. In this study, there was only a small number of respondents, 5.88% from group 1 (recognized depression) and 2.67% from group 2 (did not recognize depression), chose to ignore Linh until she gets over her problem (p < 0.05). This finding indicates that a majority of respondents believed that people with depression should not be left alone. However, 4.9% of 350 respondents with wrong knowledge about first-aid for depression was a considerable number. Young people should be educated that depressive people should not be left alone.

Concerning another component of mental health literacy—knowledge of interventions—self-help strategies (how to relax, physical exercise, consulting an online source, reading self-help book about the problem) received a high rate of endorsement by participants. Jorm and Griffiths [33] demonstrated the effectiveness of self-help strategies like exercise, relaxation and self-help book for depression. Our results also showed that the respondents in both groups believed joining a self-help group (p < 0.001) is a helpful intervention strategy. Our study again confirms that self-help strategies are essential [32] and suggests a need for public awareness campaigns on mental health problems. One interesting find in our study is that both groups (higher percentage in group 1 = 32.1% than group 2 = 26.4%) did think people with depression should be admitted to hospital for psychiatric services (p < 0.05).

A potential limitation of this study is the use of the adapted Australian questionnaire that has not been validated. However, the research team consulted mental health experts and piloted the instrument to ensure the questionnaire is understandable and relevant to the Vietnamese context. Another limitation is that our sample is a convenience one consisting of college students only. The findings are therefore not generalizable to all young adults in Vietnam. However, our study results could reflect an aspect of the “bigger picture” of Vietnamese young people’s MHL and thus, warrant future studies.

## Conclusion

Mental health literacy of depression among the undergraduate students surveyed in this study was not as high as in other countries. Aside from the majority of respondents choosing stress to describe the depression vignette, our findings suggest a need to improve understanding of MHL of depression, especially depression detection and how-to seek help, for undergraduate students in Vietnam. Although mental health problems have become an increasing problem across all socio-demographic groups in Vietnam, there are currently not enough interventions to educate the public about them. Various interventions can be considered including the development of short mental health courses for students. Additional information on various mental health issues can also be posted through social media and internet-based resources. Challenges remain as mental health has never been considered a high priority in Vietnam. Future studies should focus on how to effectively disseminate mental health education among young people.

## Abbreviations

GP: general practitioner
MHL: mental health literacy
SPSS: Statistical Package for Social Sciences
WHO: World Health Organization
